# 2′,4′-dihydroxychalcone alleviates inflammatory bowel disease by inhibiting NLRP3 inflammasome and modulating gut microbiota

**DOI:** 10.3389/fimmu.2026.1751218

**Published:** 2026-02-06

**Authors:** Guohui Zhang, Yixin Yao, Zhongyu Zhang, Jingqi Wang, Jian Xiao, Hua Yu, Mingqing Huang, Chun Yao, Yitao Wang, Hua Luo

**Affiliations:** 1 Macau Centre for Research and Development in Chinese Medicine, State Key Laboratory of Mechanism and Quality of Chinese Medicine, Institute of Chinese Medical Sciences, University of Macau, Macao, Macao SAR, China; 2 Engineering Research Center of Innovative Drug of Traditional Chinese and Zhuang Yao Medicine, Ministry of Education, Guangxi University of Chinese Medicine, Nanning, China; 3 State Key Laboratory of Bioactive Molecules and Druggability Assessment, Jinan University, Guangzhou, China; 4 College of Pharmacy, Fujian Key Laboratory of Chinese Materia Medica, Fujian University of Traditional Chinese Medicine, Fuzhou, China

**Keywords:** 2′,4′-dihydroxychalcone, gut microbiota, IL-1β, inflammatory bowel disease, NLRP3 inflammasome

## Abstract

**Introduction:**

*Abrus cantoniensis* Hance (ACH), an edible traditional Chinese medicinal herb, has significant anti-inflammatory activity. However, there is limited research on the molecular targets of its active ingredients and their application in inflammatory bowel disease (IBD). Nucleotide-binding domain and leucine-rich repeat protein 3 (NLRP3) inflammasome plays a crucial role in IBD.

**Methods:**

Here, we explored the inhibitory activity of the 25 main ingredients from ACH on NLRP3 inflammasome using THP-1 and J774A.1 macrophage models, and the dextran sulfate sodium salt (DSS)-induced acute ulcerative colitis mouse model was used to investigate the therapeutic potential. 16S rRNA analysis was performed for gut microbiota assessment.

**Results:**

The results demonstrated that 2′,4′-dihydroxychalcone (2′,4′-DHC) exhibited the remarkable inhibitory effect on the activation of NLRP3 inflammasome. ELISA and western blotting analyses revealed that 2′,4′-DHC effectively inhibited caspase-1 and Gasdermin D activation and IL-1β release but not TNF-α in macrophages. Furthermore, 2′,4′-DHC significantly alleviated inflammation in IBD mice, which mitigated body weight loss, reduced DAI score, preserved colon length and protected the gut barrier by enhance the tight junction proteins occludin and ZO-1 expression. Importantly, 2′,4′-DHC treatment inhibited NLRP3 inflammasome, while also balancing gut microbiota in colitis mice. The results showed that 2′,4′-DHC reduced the abundance of Proteobacteria, reshaped the bacterial diversity and composition.

**Discussion:**

Overall, this study identified 2′,4′-DHC in ACH that regulated the NLRP3 inflammasome activation to exert anti-inflammatory effects in IBD, highlighting its potential in treating NLRP3-related inflammatory diseases.

## 1 Introduction

*Abrus cantoniensis* Hance (ACH) is a traditional herbal medicine from the Lingnan region of China (1). Clinically, it is mainly used to treat liver-related diseases such as hepatitis (2), and also commonly prepared as herbal tea or soup (3). Since the 1960s, Chinese researchers have investigated the chemical components of ACH (4). Research has shown that ACH has multiple biological activities, such as liver protection and anti-inflammatory effects, attributed to its rich chemical composition (5, 6). Phytochemical and pharmacological activity studies have identified its active and abundant ingredients, including alkaloids (e.g., abrine and hypaphorine), flavonoids (e.g., isoshaftoside, schaftoside, and 2′,4′-dihydroxychalcone [2′,4′-DHC]), triterpenoids (e.g., ursolic acid, soyasapogenol A), and polysaccharides (7). However, most studies on its total extracts have not analyzed the chemical components, limiting understanding of its main active components, its pharmacological mechanism, and its potential application in other diseases (7).

ACH’s anti-inflammatory activity has been widely verified. Chronic inflammation, such as inflammatory bowel disease (IBD), can result from barrier dysfunction, where danger signals induce inflammasome activation, and the inflammasome-processed cytokine interleukin-1β (IL-1β) then contributes to further barrier dysfunction, dysbiosis, and the recruitment of inflammatory cells (8, 9). Nucleotide-binding domain and leucine-rich repeat protein 3 (NLRP3) inflammasome is a protein complex of NLRP3, apoptosis-associated speck-like protein containing a caspase recruitment domain (ASC), and caspase-1. Upon activation, caspase-1 induces the maturation of IL-1β and IL-18, and triggers pyroptosis by cleaving Gasdermin D (10). The activation of the NLRP3 inflammasome plays an important role in regulating the intestinal microbiota and inflammatory disorders (11–13). In IBD, the activation of NLRP3 inflammasome mainly occurs in innate immune cells, such as monocytes and macrophages, which can release most of the IL-1β from lamina propria macrophages, that are closely related to the pathogenesis of IBD (8, 9).

Although previous studies have confirmed that ACH extracts exhibited remarkable anti-inflammatory activity, and NLRP3 inflammasome is a crucial reason for amplifying intestinal inflammation in IBD, no research has yet identified the specific active ingredients and molecular targets of ACH in treating IBD (7). It remains unclear whether ACH contains compounds that directly regulate the NLRP3 inflammasome in IBD. Mouse J774A.1 and human THP-1 cells are very mature and stable cell models for inflammasome studies (14, 15). In this study, we first explored the inhibitory activity of the 25 main ingredients in ACH on NLRP3 inflammasome using THP-1 and J774A.1 macrophages. Through *in vitro* screening, we identified 2′,4′-DHC as the key active component in ACH, and for the first time confirmed that it alleviated DSS-induced colitis through a dual pathway of inhibiting NLRP3 inflammasome activation and regulating gut microbiota. This provides a clear material basis and molecular mechanism for the anti-inflammatory activity of ACH in IBD and showing great potential for developing novel therapeutic NLRP3 inhibitors.

## 2 Materials and methods

### 2.1 Reagents

Phorbol myristate acetate (PMA) (#P1585-1MG), LPS (#L4391-1mg), and ATP (#tlrl-atpl-1g) were obtained from Sigma. Nigericin (#S6653) was from Selleck. DSS (36–50 kDa) (#160110) was purchased from MP Biomedical. All ELISA kits were bought from BioLegend. RNA isolation kits were purchased from Vazyme. RT-qPCR Reagents were obtained from Takara. The sources of all the antibodies used are as follows: NLRP3 (#15101S), Caspase-1 (#83383S), Cleaved caspase-1 (#4199S and #89332S), Gasdermin D (#39754S), Cleaved Gasdermin D (#36425S and #10137S), ASC (#67824S), β-Actin (#4970S), GAPDH (#5174S), IκBα(#4814), Phospho-IκBα (#2859), and Phospho-NF-κB p65 (#3033), pro-IL-1β (#12242), IL-1β (#83184 and #63124) antibodies were purchased from Cell Signaling Technology. NF-κB p65 (#GB11142) was purchased from Servicebio. ASC antibody (#sc-514414, Santa Cruz). ZO-1(#21773-1-AP), Occludin (#27260-1-AP) and Tubulin (#11224-1-AP) antibodies were purchased from Proteintech. All the information of tested compounds were listed in **Supplementary Table S1**, including CAS number, supplier name, and catalog number, and HPLC, MS, and NMR have identified, the purity is ≥ HPLC 98%.

### 2.2 Cell culture

J774A.1 cells (SAM YAO HONG, China) were cultured in DMEM medium supplemented with 10% FBS and 1% penicillin-streptomycin. RPMI 1640 medium supplemented with 10% FBS was used to culture THP-1 cells (University of Macau, China).

To stimulate NLRP3 inflammasome activation, THP-1 cells were plated in 6-well plates with PMA (10 ng/mL) overnight, followed by LPS (500 ng/mL) treatment for 3 h. Cells were then treated with the compounds for 1 h before co-incubating with Nigericin (10 μM, 1 h) or ATP (5 mM, 1 h). J774A.1 cells were plated in 6-well plates for 24 h before being primed with LPS (1 μg/mL) overnight. Cells were then treated with the compounds for 1 h and co-incubated with Nigericin (10 μM, 1 h) or ATP (5 mM, 1 h).

### 2.3 Cell counting kit-8 assay

THP-1 cells were plated in 96-well plates and treated with PMA (10 ng/mL) overnight, followed by LPS (1 μg/mL) treatment for 3 h. Afterward, the compound was added for 2 h or 24 h. J774A.1 cells were plated in 96-well plates, treated with LPS (1 μg/mL) overnight, and then exposed to the compound for 2 h. After treatment, CCK-8 solution was added for 1–2 h, and the absorbance was measured at 450 nm.

### 2.4 ELISA

The inflammatory factors IL-6, IL-1β, and TNF-α in cell supernatants or colon homogenate were detected using ELISA kits, following the manufacturer’s protocols.

### 2.5 Western blotting

Tissue or cell proteins were separated using SDS-PAGE and transferred to PVDF membrane (#ISEQ00010, 0.2μm). The membrane was blocked with 5% nonfat dry milk for 1 h at room temperature and then incubated with different primary antibodies overnight at 4 °C. Following five washes with TBST buffer, the membranes were incubated with secondary antibodies at room temperature for 1–2 h. Protein bands were visualized using the SuperSignal™ West Femto Maximum Sensitivity Substrate (#34095, Thermo). All the quantitative statistical results of WB proteins were independently repeated in three experiments.

### 2.6 Animal experiments

C57BL/6J male mice were obtained from Guangzhou Zhiyuan Biomedical Technology Co., Ltd. (SCXK[Yue]2021-0057, No. 44826500004459). Mice were specifically pathogen-free and housed in an IVC system with a 12-hour day-night cycle. The Academic Ethics Committee of ZunYi Medical University Zhuhai Campus approved all animal experiment protocols.

After a 7-day acclimation period, 6-week-old C57BL/6J male mice were randomly divided into four groups (n=10 per group): the control group (Control), the model group (DSS), the 2′,4′-DHC low-dose group (DSS + 2′,4′-DHC 10 mg/kg), and the 2′,4′-DHC high-dose group (DSS + 2′,4′-DHC 30 mg/kg). The selection of drug dosage was based on the preliminary experiments on drug toxicity and efficacy in mice. To minimize the number of mice used as much as possible, we ultimately chose two doses of 10 and 30mg/kg for the experiments. The DSS group received oral administration of a 2.5% DSS (w/v) water solution for 7 days (Day 4–10). 2′,4′-DHC was diluted with 0.4% sodium carboxymethylcellulose for intragastric administration from Day 1–10. The body weight of the mice was recorded daily, and the disease activity index (DAI) was scored according to body weight loss, stool consistency, and fecal bleeding. Each indicator was scored from 0 to 4. On day 11, the mice were sacrificed by cervical dislocation, colon tissues were collected for photography and length measurement, and other organs were prepared for fixation or other analysis.

### 2.7 Hematoxylin-eosin, immunohistochemical, and immunofluorescence staining analysis

The tissue fixed with paraformaldehyde was paraffin-embedded and sectioned. H&E staining of tissue was performed for histological analysis. For IHC analysis, colon tissue sections were stained with NLRP3 antibody (GB114320, Servicebio) and IL-1β antibody (bs-0812R, Bioss). For IF analysis, colon tissue sections were stained with Occludin antibody (conjugated with AlexaFluor-488, Proteintech), ZO-1 antibody (conjugated with Cy3, Proteintech), and the nuclei were stained with DAPI.

### 2.8 RT-qPCR

Total RNA was isolated using the kit, and then 1 μg total RNA was transcribed into cDNA. RT-qPCR was performed following the manufacturer’s protocol. GAPDH was used as an internal control.

### 2.9 Gut microbiota analysis

16S rRNA gene sequences were obtained from mouse feces, and the diversity of gut microbiomes was analyzed using the Medical Microbial Diversity QIIME2 process Cloud Platform (Majorbio Bio-Pharm Technology Co., Ltd., Shanghai, China). Based on the SILVA 16S rRNA gene database (v138), taxonomic analysis of ASVs was performed using the Naive Bayes classifier in Qiime2. Beta diversity was analyzed by principal coordinates analysis (PCoA) and non-metric multidimensional scaling (NMDS) based on bray-curtis dissimilarity using R-3.3.1 (vegan). The raw data were uploaded to the NCBI SRA database (SRP659890).

### 2.10 Statistical analysis

All experiment data were presented as mean ± SD of three or more independent experiments. One-way analysis of variance was used for statistical evaluation through GraphPad Prism 9 software. The levels of significance were set as *p < 0.05, **p < 0.01, ***p < 0.001.

## 3 Results

### 3.1 Screening of chemical components of ACH for inhibiting IL-1β secretion in THP-1-derived macrophages

**Figure 1:**
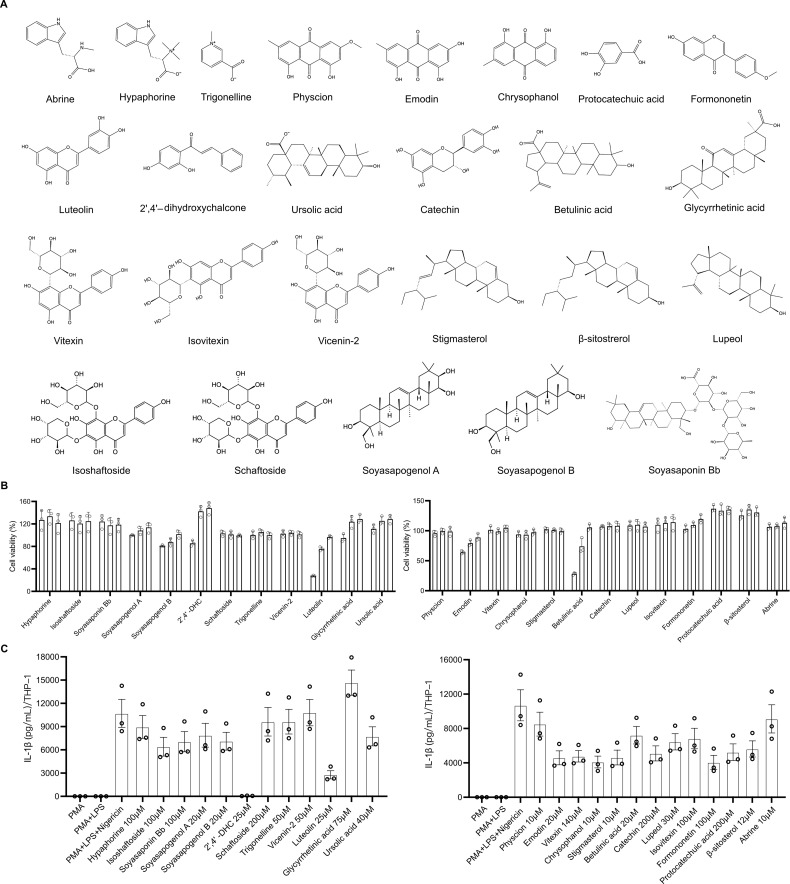
Screening of chemical components of ACH for cell viability and IL-1β secretion in THP-1-derived macrophages. **(A)** Structural formulas of 25 chemical compounds in ACH. **(B)** THP-1-derived macrophages were treated with each compound at various concentrations (the concentrations of each compound were arranged from high to low according to the **Supplementary Table S1**). CCK-8 assay was used to measure the cell viability (n = 3). **(C)** THP-1-derived macrophages were stimulated with 1 μg/mL LPS for 3 h, treated with each compound at the maximum non-toxic concentration for 1 h, and then added Nigericin for 1 h. The supernatant was collected, and ELISA was used to detect the secretion of IL-1β (n = 3).

Based on existing studies, we selected several main active ingredients in ACH, including alkaloids, triterpenes, and flavonoids (16–19). As shown in **Figure 1A**, 25 potentially active compounds were identified by UPLC, MS and NMR, including 9 flavonoids, 7 triterpenoids, 3 alkaloids, 3 anthraquinones, 2 sterols, and 1 organic acid. THP-1-derived macrophages were treated with these components for 24 h to determine the highest non-cytotoxic concentrations by CCK-8 (**Figure 1B**), and the concentrations of each compound used were provided in **Supplementary Table S1**. Nigericin was added to boost human IL-1β release. The results showed that among the compounds tested, 2′,4′-DHC markedly inhibited IL-1β secretion at 25 μM in THP-1 (**Figure 1C**). Given its effective lower concentration and potent inhibitory activity of IL-1β release, 2′,4′-DHC was chosen for further investigation.

### 3.2 2′,4′-DHC inhibited NLRP3 activation by inhibiting caspase-1 and Gasdermin D cleavage

**Figure 2:**
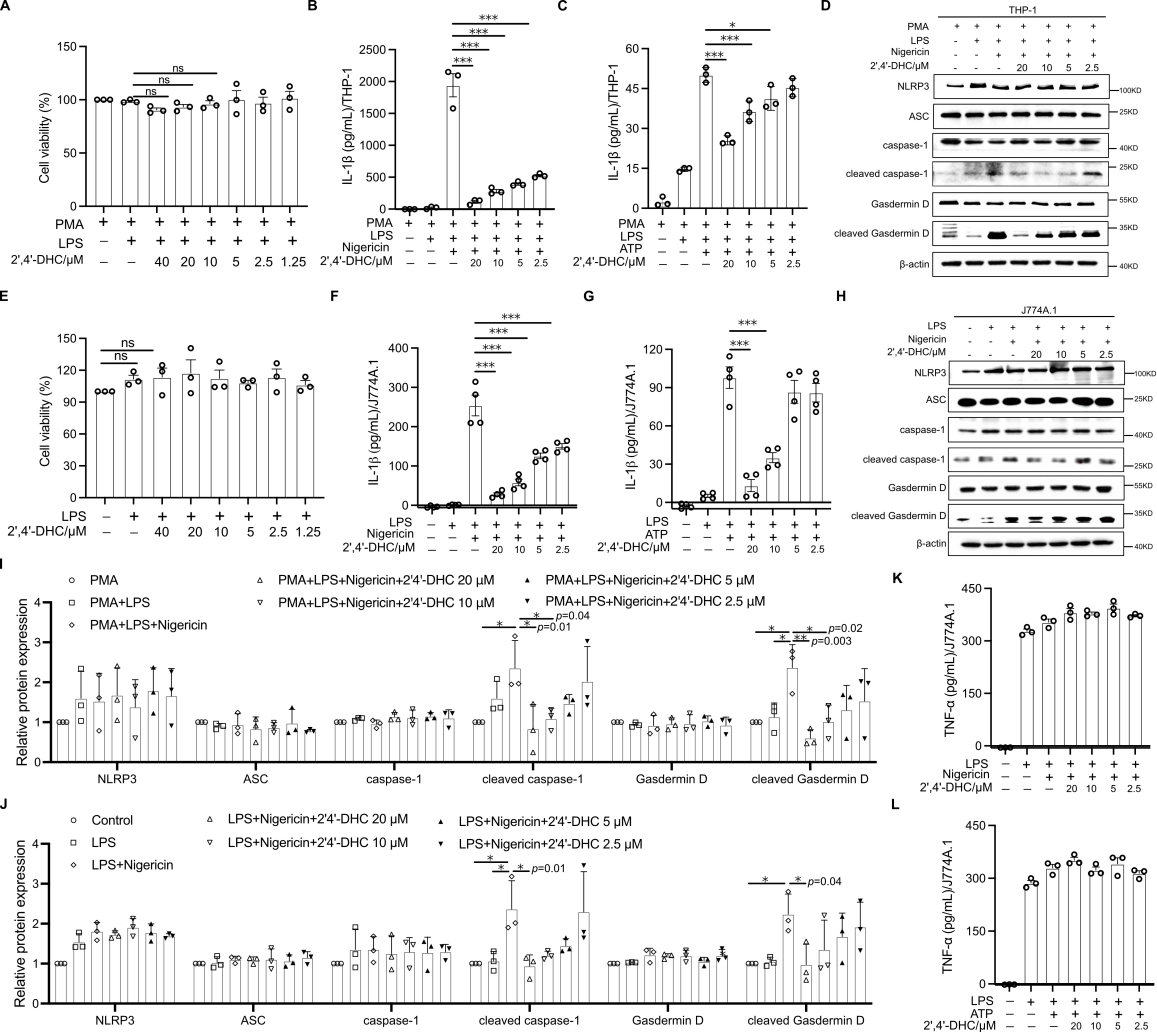
2′,4′-DHC inhibited NLRP3 activation. **(A)** THP-1-derived macrophages stimulated with 0.5 μg/mL LPS for 3 h, 2′,4′-DHC was treated for 2 h CCK-8 assay was used to measure cell viability. ELISA analysis of IL-1β of THP-1-derived macrophages which were boosted by Nigericin **(B)** or ATP **(C)** (n = 3). The expressions of NLRP3, ASC, caspase-1, cleaved caspase-1, Gasdermin D, and cleaved Gasdermin D were detected by WB in lysates of THP-1 cells **(D)** and J774A.1 cells **(H)**. Quantitative analysis of protein levels of THP-1 cells **(I)** and J774A.1 cells **(J)** (n=3). **(E)** LPS-primed J774A.1 cells were incubated with 2′,4′-DHC for 2 h, and CCK-8 assay was used to measure the cell viability. LPS-primed J774A.1 cells were incubated with 2′,4′-DHC for 1 h and then incubated with Nigericin **(F, K)** or ATP **(G, L)** for 1h. ELISA analysis of IL-1β **(F, G)** or TNF-α **(K, L)** in the supernatant (n=3–4). Data are presented as mean ± SD. *p < 0.05, **p < 0.01, ***p < 0.001, *ns* not significant.

To test whether 2′,4′-DHC affects the activation of the NLRP3 inflammasome in human and mouse macrophages, we established NLRP3 inflammasome activation models using the human peripheral blood mononuclear cell line THP-1, which can differentiate into human macrophages (20), and the mouse macrophage cell line J774A.1 (14). No cytotoxicity of 2′,4′-DHC to THP-1-derived macrophages and J774A.1 macrophages at concentrations below 40 μM (**Figures 2A, E**). Nigericin and ATP can induce IL-1β production, a marker activated by the NLRP3 inflammasome (21). The results demonstrated that 2′,4′-DHC exhibited remarkable concentration-dependent inhibitory effects on IL-1β secretion (**Figures 2B, C, F, G**). In addition, we found that 2′,4′-DHC did not affect pro-IL-1β expression, but only inhibited the production of IL-1β in supernatant (**Supplementary Figures S1A, B**).

Caspase-1 activation by NLRP3 inflammasome is essential for maturation of IL-1β during the innate immune response. And Gasdermin D activation is necessary for IL-1β release. Here the WB results showed, 2′,4′-DHC significantly inhibited the expression of cleaved caspase-1 and cleaved Gasdermin D without affecting the expression levels of NLRP3, ASC, caspase-1 and Gasdermin D (**Figures 2D, I, H, J**). To test the specificity of 2′,4′-DHC’s inhibitory effect on NLRP3 activation, we evaluated its impact on TNF-α release, which occurs independently of NLRP3 inflammasome activation (21). TNF-α release was induced by LPS treatment with or without Nigericin or ATP, and 2′,4′-DHC showed no inhibitory effect on TNF-α release (**Figures 2K, L**). Furthermore, 2′,4′-DHC did not affect the phosphorylation levels of p65 and IκB (**Supplementary Figure S2**). Also, it did not inhibit NLRP3 protein levels (**Figures 2D, H**), pro-IL-1β expression (**Supplementary Figure S1**) and TNF-α release (**Figures 2K, L**). These findings demonstrated that 2′,4′-DHC selectively inhibited NLRP3 activation by inhibiting caspase-1 and Gasdermin D cleavage in both human and mouse macrophages, not by inhibiting the upstream NF-κB pathway.

### 3.3 2′,4′-DHC showed significant efficacy against DSS-induced colitis in mice

**Figure 3:**
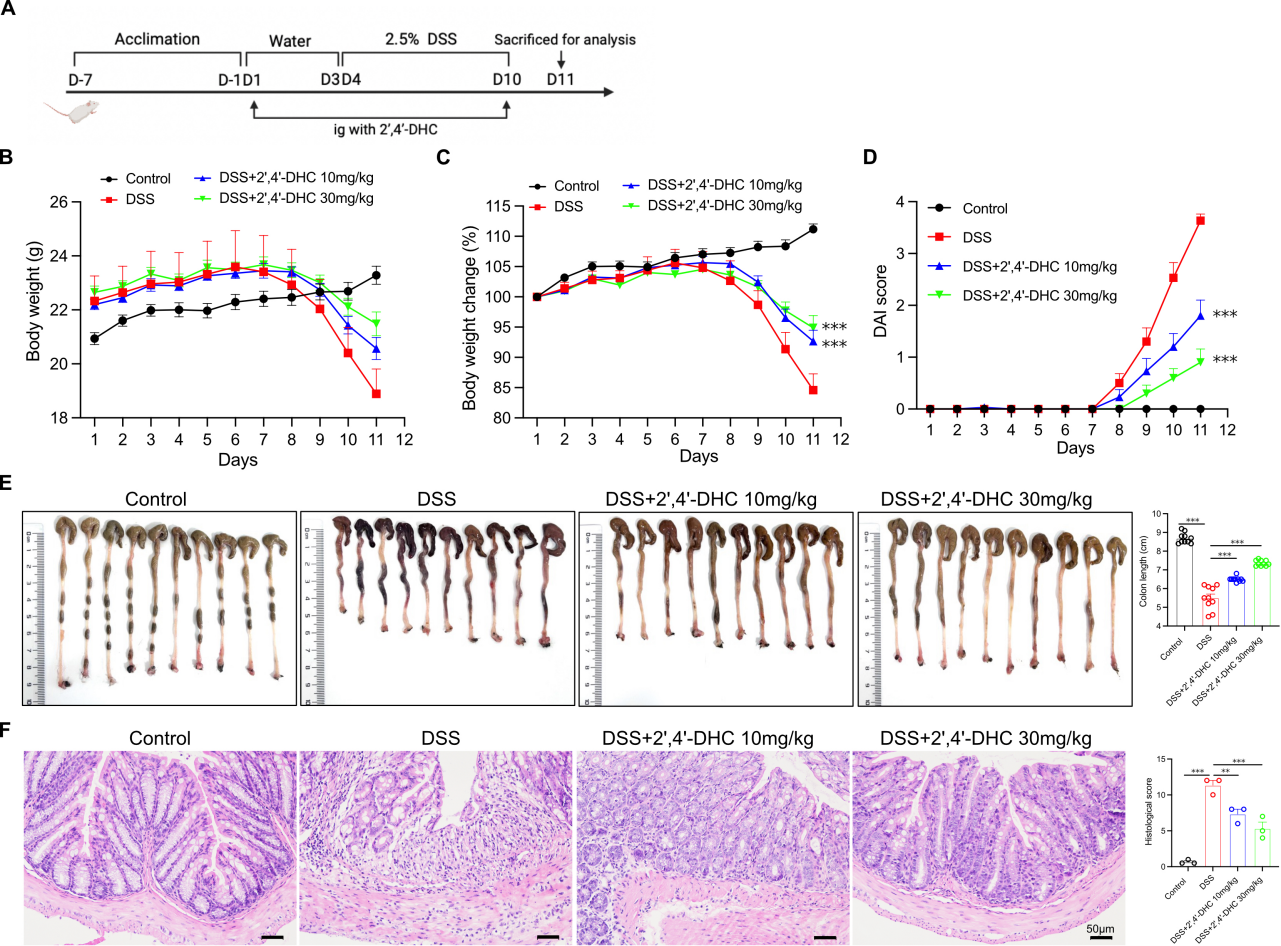
2′,4′-DHC showed significant efficacy against DSS-induced colitis in mice. **(A)** Schematic flow diagram of animal experiment procedure. **(B)** Body weight and **(C)** Body weight change of each group. (n=10) **(D)** DAI score. (n=10) **(E)** Photos of colon morphology of each sample and the average lengths of each group. (n=10) **(F)** H&E stained colon sections (scale bar =50 μm) and the histopathological score of each group (n=3). Data are presented as mean ± SD. **p < 0.01, ***p < 0.001, compared to the DSS group.

Given the crucial role of NLRP3 inflammasome activation in the pathogenesis of colitis (8), we evaluated the therapeutic potential of 2′,4′-DHC using a DSS-induced acute ulcerative colitis mouse model (**Figure 3A**). Treatment with 2′,4′-DHC significantly mitigated body weight loss in mice (**Figures 3B, C**), reduced DAI score (**Figure 3D**), and preserved colon length (**Figure 3E**) compared to the DSS group. Histological analysis revealed mucosal ulceration, crypt loss, and epithelial damage in colon tissue of the DSS group, whereas 2′,4′-DHC treatment significantly alleviated these pathological injuries and reduced the histological score (**Figure 3F**). These findings indicated that 2′,4′-DHC exerted significant therapeutic effects against DSS-induced colitis in mice.

### 3.4 2′,4′-DHC inhibited NLRP3 inflammasome activation in mice with DSS-induced colitis

**Figure 4:**
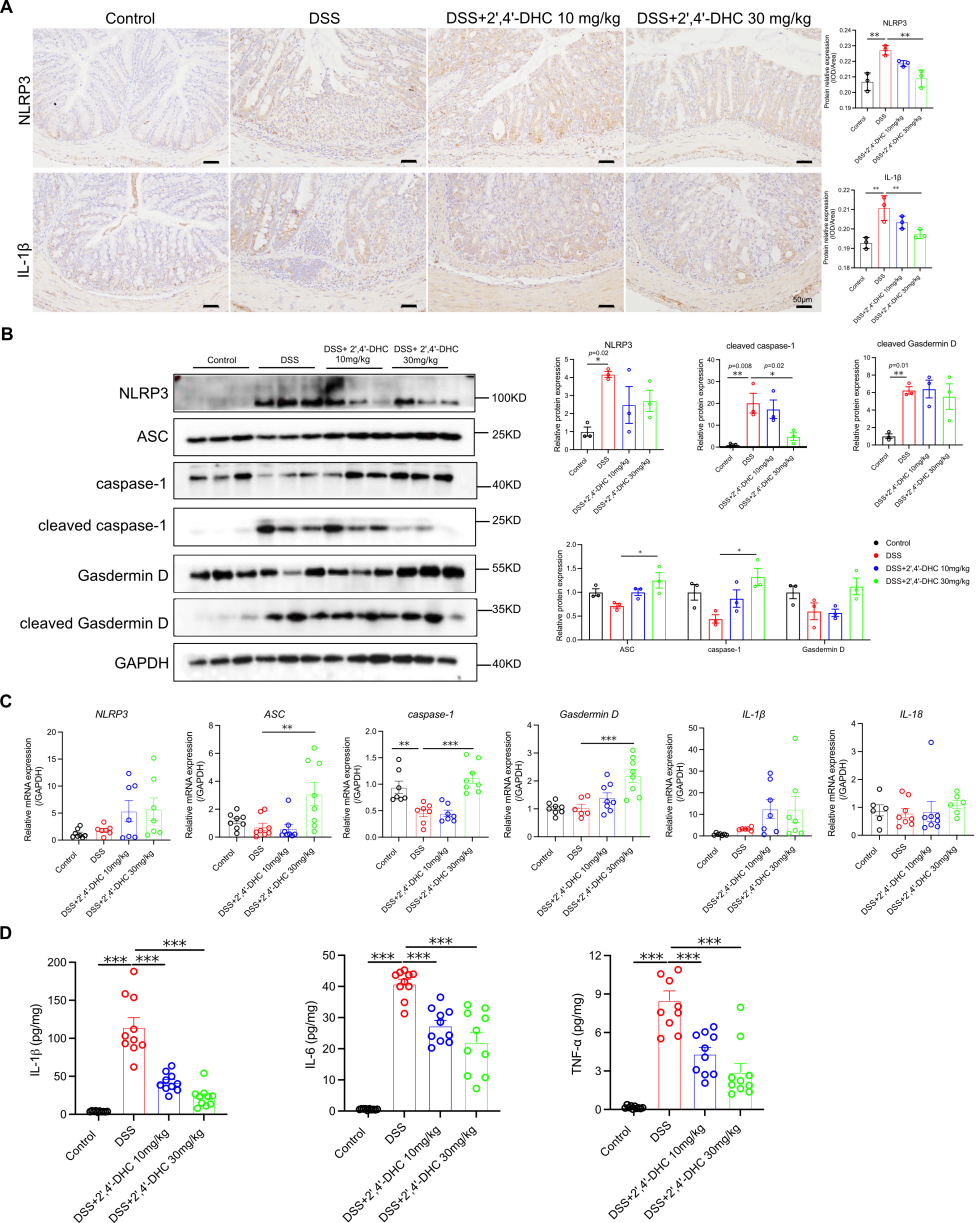
2′,4′-DHC inhibited NLRP3 activation in DSS-induced colitis model. **(A)** IHC analysis of NLRP3 and IL-1β in colon, scale bar =50 μm. IOD/Area values were measured by ImageJ (n=3) **(B)** WB analysis of NLRP3, ASC, caspase-1, cleaved caspase-1, Gasdermin D, and cleaved Gasdermin D in the colon (n=3). **(C)** Relative mRNA expression of NLRP3, ASC, caspase-1, Gasdermin D, IL-1β, and IL-18 in the colon (n=6–8). **(D)** The expression of IL-1β, IL-6, and TNF-α in colonic tissue homogenate (n=10). Data are presented as mean ± SD. *p < 0.05, **p < 0.01, ***p < 0.001, compared to the DSS group.

We further investigated whether 2′,4′-DHC had an inhibitory effect on the activation of NLRP3 inflammasome *in vivo*. DSS-induced acute intestinal inflammation in mice led to significant activation of the NLRP3 inflammasome, as evidenced by increased NLRP3, cleaved caspase-1, cleaved Gasdermin D expression, and pro-inflammatory factors IL-1β, IL-6, and TNF-α. Treatment with 2′,4′-DHC effectively inhibited these changes (**Figures 4A, B, D**). Interestingly, the 2′,4′-DHC treatment showed increased mRNA and protein levels of ASC, caspase-1 and Gasdermin D (**Figures 4B, C**). In the process of regulating the activity of the NLRP3 inflammasome, multiple proteins need to interact, and it involves different stages of activation, which is a stepwise activation process. Therefore, it is very likely that there is a negative feedback regulation. We have also found similar results in other studies (14, 22). This increase might be attributed to 2′,4′-DHC’s inhibition of NLRP3 inflammasome activation, leading to the accumulation of related proteins, especially caspase-1, which requires further in-depth verification. These findings demonstrated that 2′,4′-DHC primarily inhibited NLRP3 inflammasome activation by inhibiting caspase-1 activation in DSS-induced colitis.

### 3.5 Effects of 2′,4′-DHC on the gut barrier, gut microbiota, and safety evaluation

**Figure 5:**
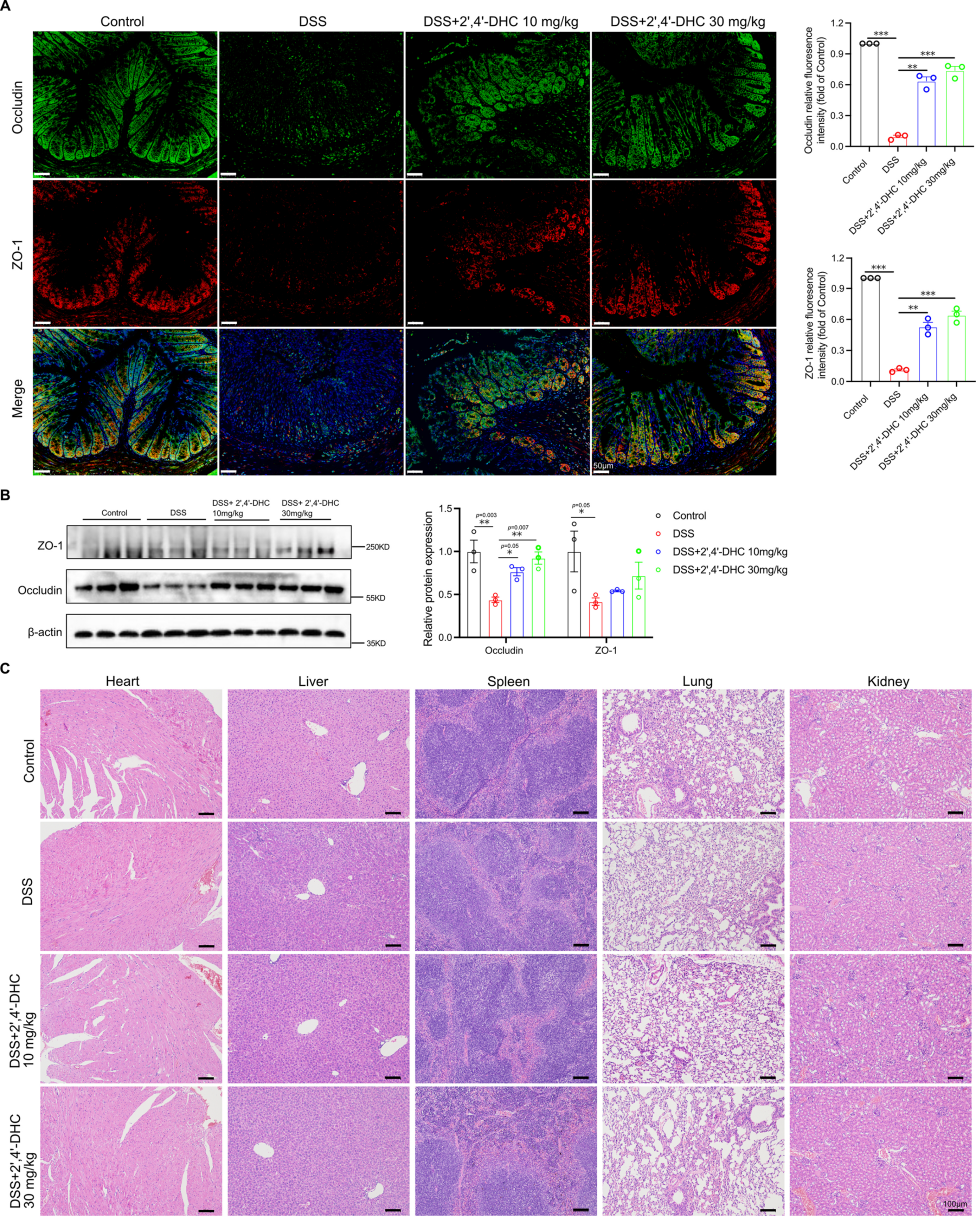
Effects of 2′,4′-DHC on the gut barrier function and safety evaluation. **(A)** IF analysis and **(B)** WB analysis of tight junction proteins ZO-1 and occludin expression in each group, scale bar=50 μm. (n=3) **(C)** H&E staining of main internal organs (heart, liver, spleen, lung, and kidney), scale bar=100 μm. Data are presented as mean ± SD. *p < 0.05, **p < 0.01, ***p < 0.001, compared to the DSS group.

We further assessed the effects of 2′,4′-DHC on the gut barrier, gut microbiota, and its safety. 2′,4′-DHC effectively increased the expression of tight junction proteins occludin and ZO-1 in the colon compared to the DSS group (**Figures 5A, B**), indicating its role in restoring the gut barrier integrity. Histological examination of major organs from each group of mice revealed no obvious abnormal lesions (**Figure 5C**), indicating that 2′,4′-DHC exerted its effect of restoring intestinal barrier integrity without causing damage to major organs.

**Figure 6:**
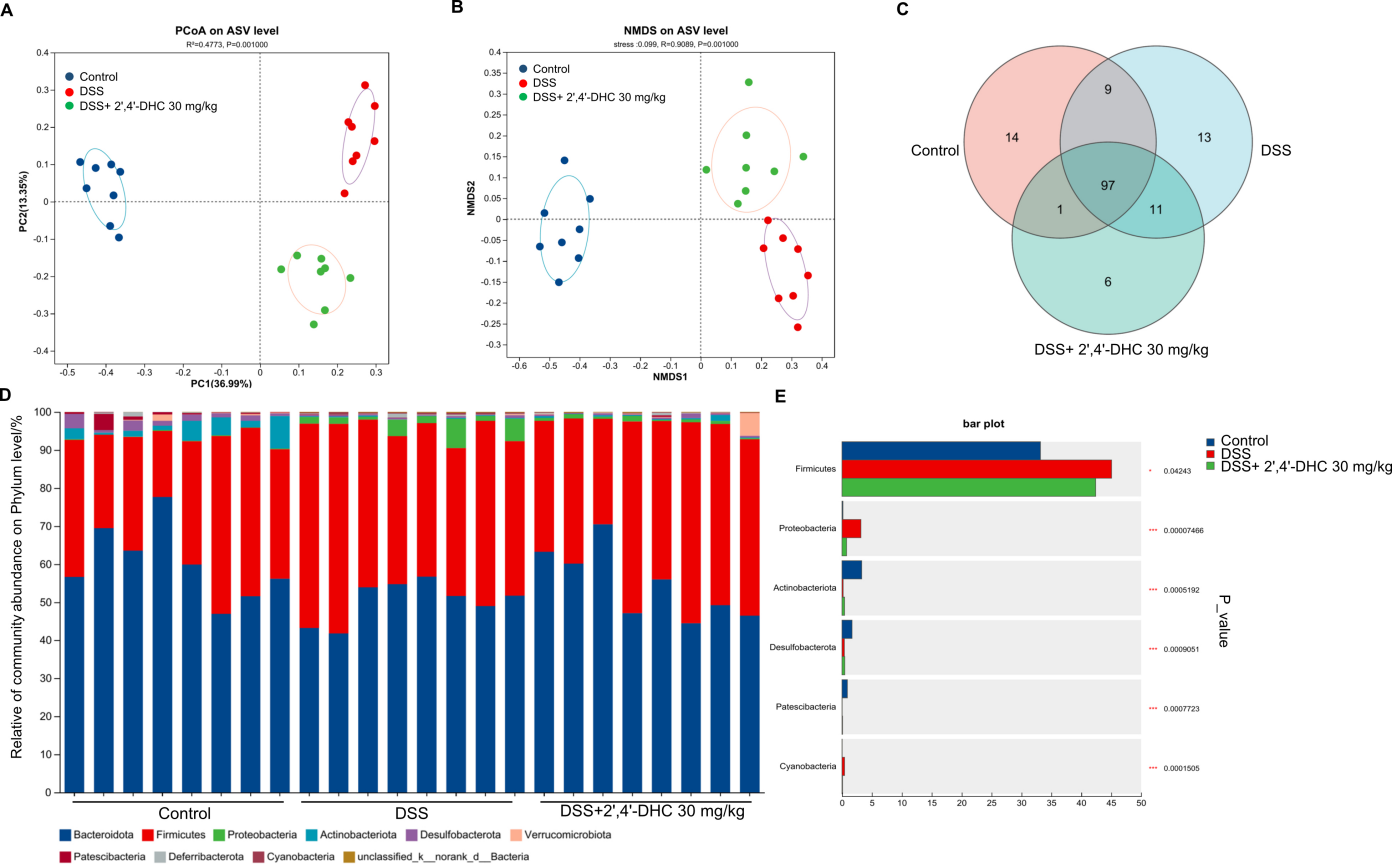
2′,4′-DHC modulated gut microbial diversity and composition in mice with colitis. **(A)** PCoA on ASV level. (n=8) **(B)** NMDS on ASV level. (n=8) **(C)** Venn diagram. **(D)** Percent of community abundance on phylum level. **(E)** Abundance of community at the phylum level in three groups. (n=8).

Increasing research has confirmed that gut microbiota dysbiosis is closely related to IBD, characterized by a decrease in beneficial bacteria and an increase in opportunistic pathogenic bacteria, such as *Proteobacteria* (23). To further investigate this relationship, we analyzed mouse fecal bacteria by performing high-throughput gene sequencing analysis of 16S rRNA. Beta diversity analysis of PCoA (**Figure 6A**) and NMDS (**Figure 6B**) results showed a significant separation of 2′,4′-DHC treatment from the Control and the DSS groups. The Venn diagram showed that there were 6 unique and 97 common taxa in the 2′,4′-DHC treatment group (**Figure 6C**). Subsequently, the community barplot analysis at the phylum level revealed an increase in the *Proteobacteria* proportions in the DSS group. The abundance of *Proteobacteria* is considered as the signature of dysbiosis in gut microbiota (24). After 2′,4′-DHC treatment, the abundance of *Proteobacteria* significantly reduced in mice with colitis (**Figures 6D, E**).

**Figure 7:**
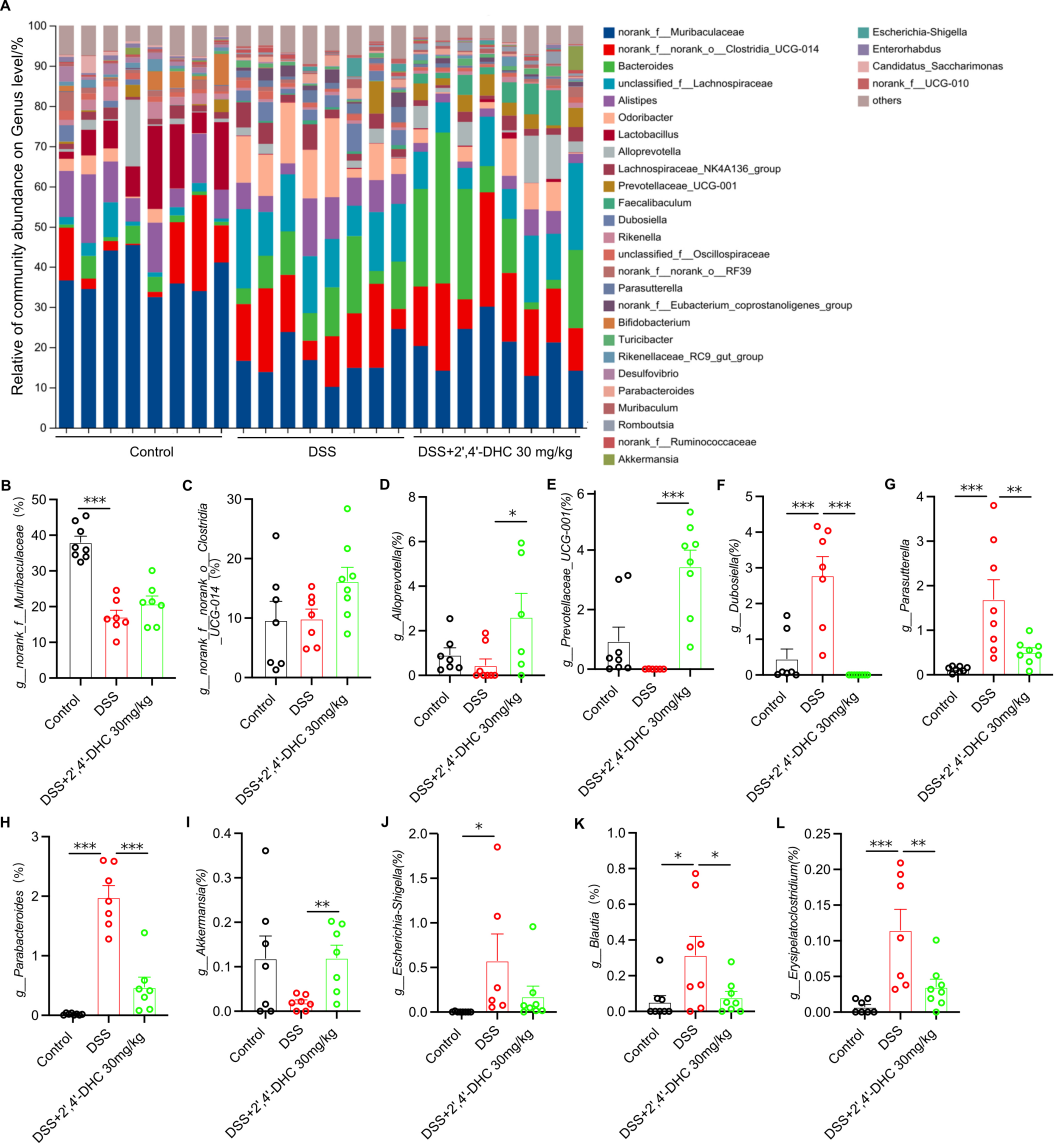
Gut microbial changes at the genus level. **(A)** Community barplot analysis at the genus level. Relative abundance of **(B)***Muribaculaceae*, **(C)***Clostridia_UCG-014*, **(D)***Alloprevotella*, **(E)***Prevotellaceae_UCG-001*, **(F)***Dubosiella*, **(G)***Parasutterella*, **(H)***Parabacteroides*, **(I)***Akkermansia*, **(J)***Escherichia-Shigella*, **(K)***Blautia*, and **(L)***Erysipelatoclostridium* (n=6–8). Data are presented as mean ± SD, *p < 0.05, **p < 0.01, ***p < 0.001, compared with the DSS group.

Furthermore, we also observed remarkable changes in gut microbiota at the genus level (**Figure 7A**), the relative abundance of *norank_f_Muribaculaceae*, *norank_f_norank_o_Clostridia_UCG-014*, *Alloprevotella*, *Prevotellaceae_UCG-001*, and *Akkermansia* improved after 2′,4′-DHC treatment (**Figures 7B–E, I**). Meanwhile, 2′,4′-DHC treatment reduced DSS-induced increase in certain microbial populations, including *Dubosiella*, *Parasutterella*, *Parabacteroides*, *Escherichia-Shigella*, *Blautia*, and *Erysipelatoclostridium* (**Figures 7F–H, J–L**). Research have found that *Dubosiella*, *Parasutterella*, *Erysipelatoclostridium*, and *Escherichia-Shigella* were significantly increased (23, 25, 26), while *norank_f_Muribaculaceae* and *Prevotellaceae_UCG-001* decreased after DSS treatment (25), which were consistent with our findings. Meanwhile, we identified the specific gut microbes after 2′,4′-DHC treatment by LEfSe. The results confirmed that 2′,4′-DHC enriched beneficial taxa, *Bacteroides* and *Bacteroidaceae* (**Supplementary Figures 3SA, B**). Amino acid metabolism was significant changed in 2′,4′-DHC group (**Supplementary Figure 3SC**), and which was essential in protein synthesis of intestinal microbiota (26).

## 4 Discussion

The ambiguity of the main active ingredients of ACH has limited its clinical application. Here, we demonstrated that several chemical components of ACH, including luteolin, emodin, chrysophanol, and formononetin, exhibited NLRP3 inhibitory activity, laying a material foundation for its potential clinical application, such as IBD. We found that 2′,4′-DHC exhibited remarkable anti-inflammasome activity both *in vivo* and *in vitro*. Chalcones are aromatic ketones with an α, β-unsaturated carbonyl group. Increasing research confirms that many natural chalcones or derivatives have inhibitory effects on the NLRP3 inflammasome, such as echinatin (27), abelmanihotols A (28). Structurally, they all share a common functional group, the α, β-unsaturated carbonyl group, which is found in known NLRP3 inhibitors such as Oridonin (21), and this might be one reason why they can bind to the NLRP3 protein and inhibit the activation of the NLRP3 inflammasome. Importantly, the chalcone scaffold can be modified at multiple points, with its structural diversity, synthetic feasibility, and versatility giving it great potential for developing novel NLRP3 inhibitors (29). This study discovered a natural chalcone 2′,4′-DHC that significantly inhibited NLRP3 inflammasome activation and IL-1β secretion both *in vivo* and *in vitro*, showing great potential for the treatment of IBD. Moreover, 2′,4′-DHC treatment decreased the release of inflammatory factors IL-1β, IL-6, and TNF-α, while increasing the expression of tight junction proteins ZO-1 and occludin, thereby maintaining the integrity and function of the intestinal barrier.

We also found its efficacy in modulating gut microbiota. The gut microbiota and the host generally have a mutually beneficial symbiotic relationship. Their metabolites not only maintain gut homeostasis but also help inhibit the growth of pathogenic bacteria and maintain the integrity of the intestinal barrier. Therefore, changes in the gut microbiota can affect immune regulation, triggering inflammation (30). The increase in *Bacteroidota*, such as *Muribaculaceae* and *Alloprevotella*, and decrease in *Proteobacteria*, such as *Escherichia-Shigella* restored the composition of the gut microbiome. Besides, the *Clostridia* contributed to the production of short-chain fatty acids, which induced Treg cell production by inhibiting pro-inflammatory cytokines and promoting Foxp3 transcription. This is important for regulating the TH17/Treg balance and helps maintain intestinal immune homeostasis (31). *Prevotellaceae UCG-001* is beneficial for glycolipid metabolism and the degradation of fibrous substances (32). *Akkermansia* is closely related to maintaining the thickness of the intestinal mucus layer (33). Taken together, 2′,4′-DHC regulated the gut microbiome by altering bacterial diversity and composition, which were associated with the alleviation of colitis. However, the causal relationship needs to be further verified through fecal microbiota transplantation, and it remains unknown whether its improvement in gut microbiota is dose-dependent, which is a limitation of this study.

We demonstrated that DSS significantly activated NLRP3 inflammasome, while ASC decreased at both protein and mRNA levels, consistent with findings from another study (14). NLRP3 plays a key role in recruiting ASC and caspase-1 to form an active NLRP3 inflammasome, leading to caspase-1 activation, which cleaves pro-IL-1β into IL-1β. In general, NLRP3 inflammasome has a protective effect on the intestinal commensal microbiota homeostasis and intestinal barrier at the early stages of IBD (8). When the intestinal barrier is damaged, NLRP3 inflammasome can aggravate the inflammatory response by accumulating inflammatory factors, such as TNF-α, IL-1β, and IL-6 (34), causing further colon tissue damage (8, 35) and disrupting intestinal microbiota homeostasis (36). On the one hand, Nlrp3^-/-^ and Casp1^-/-^ mice were more susceptible to DSS-induced colitis, the reason may be the deficiency of IL-18 production by NLRP3 inflammasome in epithelial cells (37), which was a crucial mediator for the mucosal barrier (38). On the other hand, the microbiota can stimulate the IL-1β release via NLRP3 inflammasome activation, which boosts immune responses and enhances inflammation in the intestine, inducing more intestinal injury (9). Therefore, controlling the overactivation of NLRP3 inflammasome is an effective treatment for alleviating IBD (8, 35).

**Figure 8:**
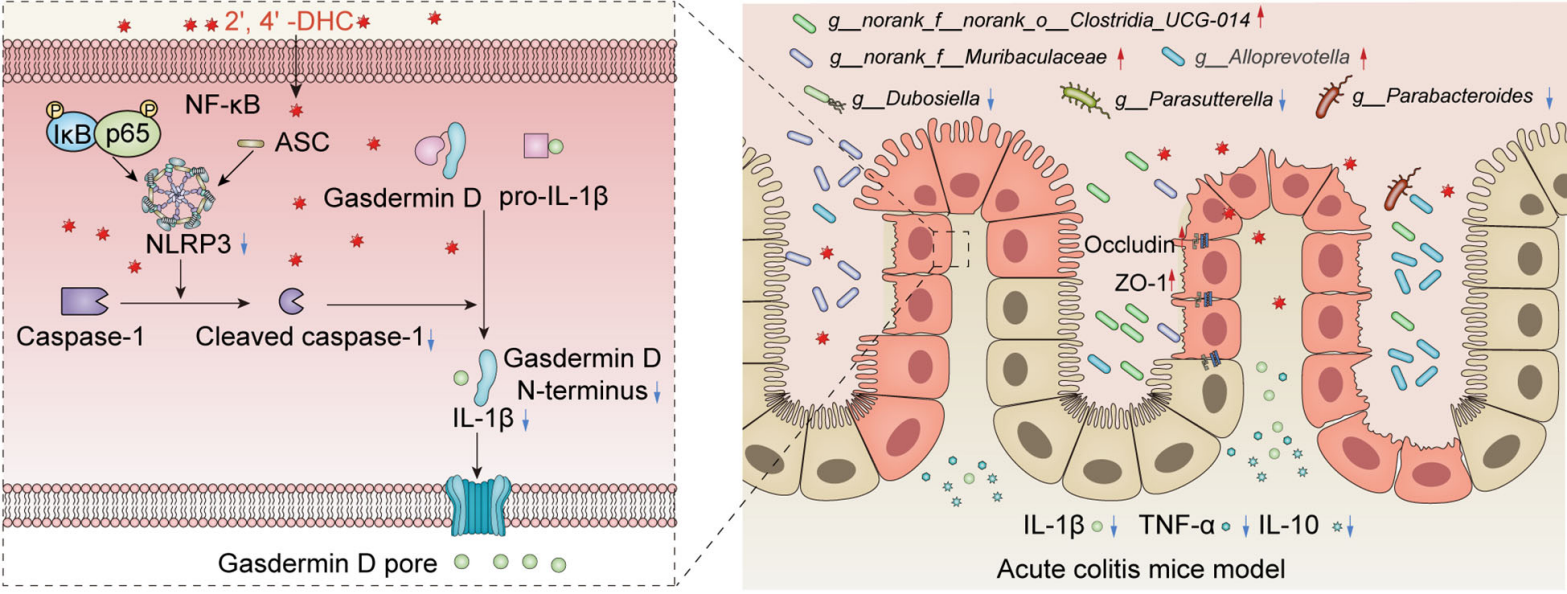
Pharmacological mechanism of 2′,4′-DHC in alleviating colitis.

IL-1β, a critical inflammatory factor in colitis, is primarily induced by NLRP3 inflammasome activation in monocytes (9). IL-1β directly triggers immune responses in the colon by stimulating T cells or neutrophils to further release other inflammatory cytokines, such as IL-6, and TNF (8). In this study, we demonstrated that 2′,4′-DHC inhibited NLRP3 inflammasome overactivation by inhibiting caspase-1 activation, thereby reducing intestinal inflammatory response and increased the homeostasis of the gut microbiota (**Figure 8**).

NLRP3 inflammasome is mainly expressed in colonic epithelial cells, subepithelial macrophages, and other inflammatory immune cells in the gut (35). The expression of NLRP3 is significantly increased during the development of colitis-associated colorectal cancer and higher levels of NLRP3 are negatively correlated with the prognosis of colorectal cancer (39). 2′,4′-DHC did not affect the expression of NLRP3 in macrophages *in vitro*, but it did inhibit the NLRP3 expression in colon tissue. Macrophages have high plasticity and are very sensitive to change in the microenvironment, they can adapt to the environment and perform various function, resulting in the diversity and heterogeneity (40, 41). Our previous study found that 2′,4′-DHC could directly bind to NLRP3, and inhibit the expression and activation of NLRP3 in colorectal cancer cells (42). The characteristic skeleton of chalcone can be modified at specific sites to yield derivatives with significant antitumor (43) and anti-inflammatory activities (44). Interestingly, the results in **Figure 2** showed that the effective concentration of 2′,4′-DHC in macrophages to inhibit NLRP3 inflammasome activation was comparable to that of in colon cancer cells to inhibit tumor cell proliferation (42). This study further confirmed that the natural chalcone 2′,4′-DHC can exert inhibitory effects in both colorectal cancer and colitis, showing great potential for developing novel therapeutic agents, especially for NLRP3 inhibitors. Given that macrophages have a significant impact on the process of colitis-associated colorectal cancer progression (45). We will continue to investigate the therapeutic potential of 2′,4′-DHC in macrophages in the future, particularly in the colitis-associated colorectal cancer model. As the mechanism of action of first-line drug-5-aminosalicylic acid for IBD (25) was inconsistent with this study, we did not add a positive control drug in animal experiments, which was also a limitation of this study.

In conclusion, our study found that the natural chalcone compound 2′,4′-DHC could be a significant potential candidate for developing NLRP3 inhibitor. 2′,4′-DHC acted as an excellent protective agent that alleviated IBD, operating through several critical mechanisms: a) inhibition of NLRP3 activation by inhibiting caspase-1 and Gasdermin D activation; b) restoring intestinal barrier integrity; and c) regulating gut microbiota balance, accompanied by inhibiting inflammation within both the colon and systemic circulation through microbe-host interactions. With practical inhibitory effect on the NLRP3 inflammasome, and comes from a green natural herbal medicine, 2′,4′-DHC emerges as an up-and-coming agent for clinical application treating IBD.

## Data Availability

The datasets presented in this study can be found in online repositories. The names of the repository/repositories and accession number(s) can be found below: SRP659890 (SRA, NCBI).
