# Supplementary material for: 2′,4′-dihydroxychalcone alleviates inflammatory bowel disease by inhibiting NLRP3 inflammasome and modulating gut microbiota
Source: Front Immunol. 2026 Feb 6;17:1751218. doi: 10.3389/fimmu.2026.1751218 (PMC12920247; doi:10.3389/fimmu.2026.1751218)

Supplementary data

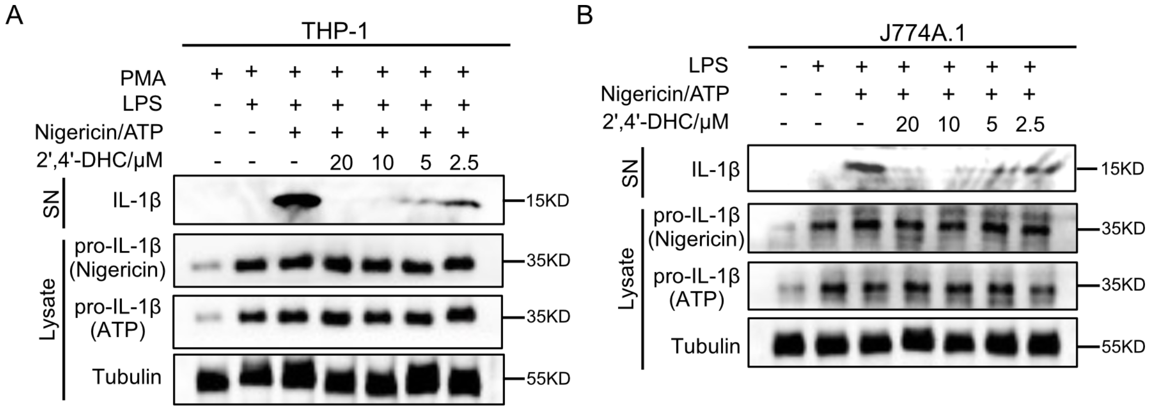

Figure S1. 2',4'-DHC inhibited IL-1β release in THP-1 and J774A.1 cells. The expressions of IL-1β in culture supernatants (SN) and pro-IL-1β in lysates were detected by WB of THP-1 cells (A) and J774A.1 cells (B).

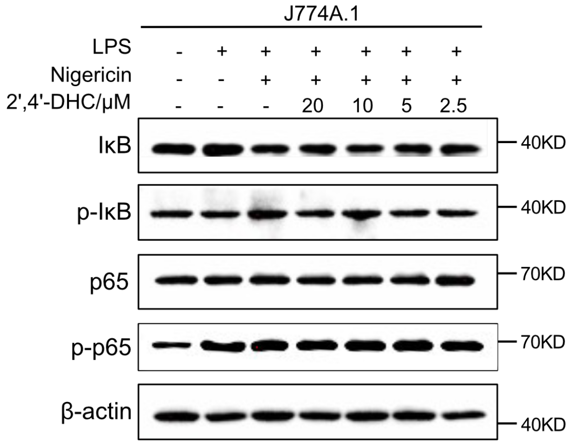

Figure S2. 2',4'-DHC did not inhibit the activation of the NF-κB pathway. IκB, p-IκB, p65, and p-p65 were detected by WB in J774A.1 cells.

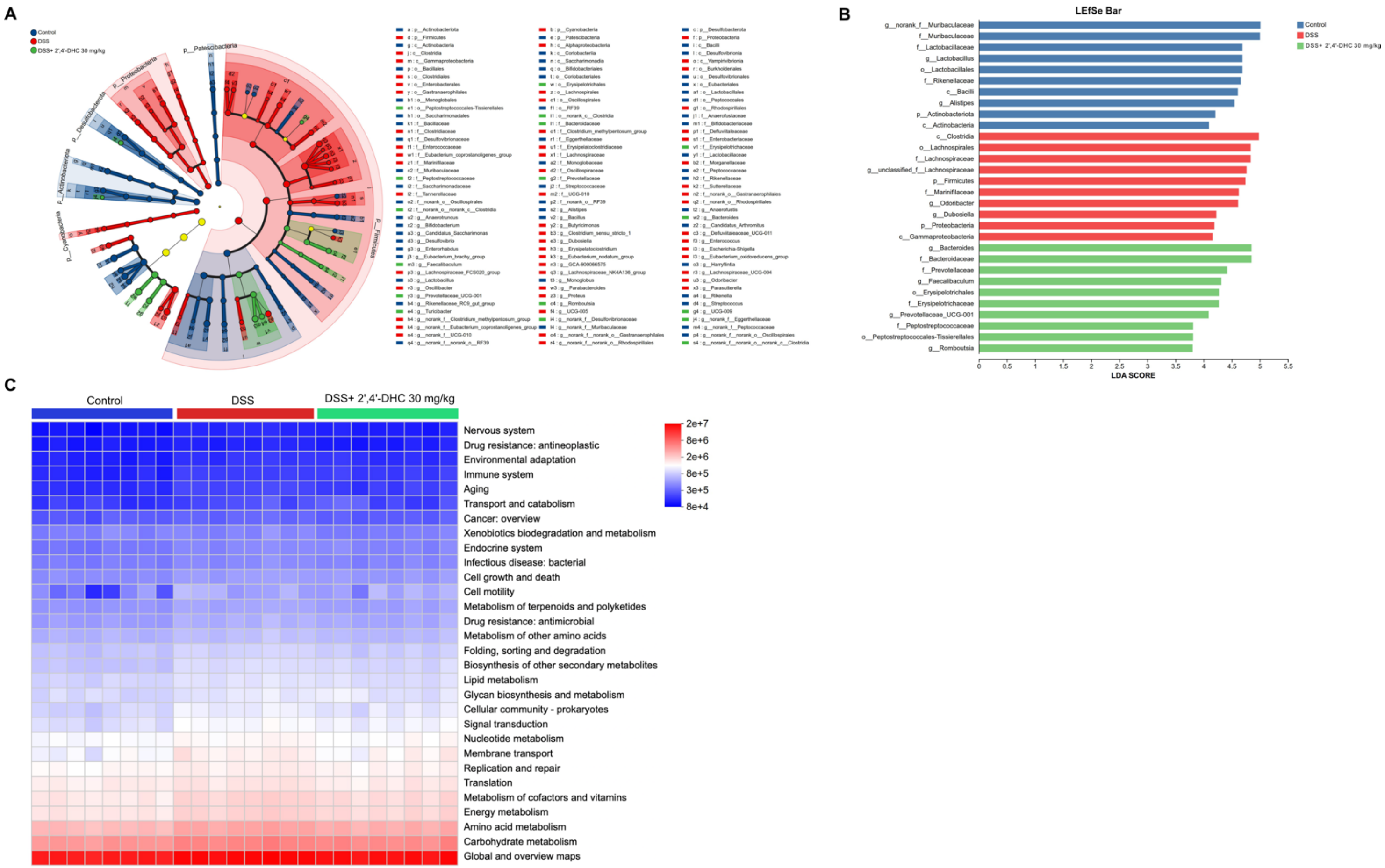

Figure S3. Linear discriminant analysis (LDA) integrated with effect size (LEfSe). (A) Cladogram indicating the phylogenetic distribution of microbiota correlated with different groups. (B) The differences in abundance among groups. (C) The differential abundance of KEGG level 2 pathways.

Table S1. The concentrations of compounds used to treat THP-1-derived macrophages

Compounds	CAS	Supplier name	Catalog number	Concentrations/μM	Safe dose/μM
Hypaphorine	487-58-1	Taihe (Guangzhou) Biotechnology Co., Ltd.	DC0089-0020	100, 50, 25	100
Isoshaftoside	52012-29-0	Taihe (Guangzhou) Biotechnology Co., Ltd.	DY0019-0020	100, 50, 25	100
Soyasaponin Bb	51330-27-9	Taihe (Guangzhou) Biotechnology Co., Ltd.	DD0025-0020	100, 50, 25	100
Soyasapogenol A	508-01-0	Taihe (Guangzhou) Biotechnology Co., Ltd.	DD0100-0010	40, 20, 10	20
Soyasapogenol B	595-15-3	Taihe (Guangzhou) Biotechnology Co., Ltd.	DD0101-0010	50, 25, 12.5	20
2',4'-DHC	1776-30-3	Chengdu Chroma-Biotechnology Co., Ltd.	CHB-E-119	50, 25, 12.5	25
Schaftoside	51938-32-0	Taihe (Guangzhou) Biotechnology Co., Ltd.	DX0006-0020	200, 100, 50	200
Trigonelline	535-83-1	Taihe (Guangzhou) Biotechnology Co., Ltd.	DH0040-0020	50, 25, 12.5	50
Vicenin-2	23666-13-9	Taihe (Guangzhou) Biotechnology Co., Ltd.	DX0006-0020	50, 25, 12.5	50
Luteolin	491-70-3	Taihe (Guangzhou) Biotechnology Co., Ltd.	DM0032-0020	100, 50, 25	25
Glycyrrhetinic acid	471-53-4	Taihe (Guangzhou) Biotechnology Co., Ltd.	DG0007-0020	100, 50, 25	75
Ursolic acid	77-52-1	Taihe (Guangzhou) Biotechnology Co., Ltd.	DX0019-0020	40, 20, 10	40
Abrine	526-31-8	Taihe (Guangzhou) Biotechnology Co., Ltd.	DX0104-0020	10, 5, 2.5	10
Physcion	521-61-9	Chengdu Push Bio-technology Co., Ltd.	PS0291-0020	10, 5, 2.5	10
Emodin	518-82-1	Chengdu Push Bio-technology Co., Ltd.	PU0062-0025	100, 50, 25	20

Vitexin	3681-93-4	Chengdu Push Bio-technology Co., Ltd.	PS0760-0020	140, 70, 35	140
Chrysophanol	481-74-3	Chengdu Push Bio-technology Co., Ltd.	PS02993-0020	10, 5, 2.5	10
Stigmasterol	83-48-7	Chengdu Push Bio-technology Co., Ltd.	PS0773-0020	10, 5, 2.5	10
Betulinic acid	472-15-1	Chengdu Push Bio-technology Co., Ltd.	PS0348-0020	75, 37.5, 18.75	20
Catechin	154-23-4	Chengdu Push Bio-technology Co., Ltd.	PU0728-0025	200, 100, 50	200
Lupeol	545-47-1	Chengdu Chroma-Biotechnology Co., Ltd.	CHB-Y-153	30, 15, 7.5	30
Isovitexin	38953-85-4	Chengdu Chroma-Biotechnology Co., Ltd.	CHB-Y-105	100, 50, 25	100
Formononetin	485-72-3	Chengdu Chroma-Biotechnology Co., Ltd.	CHB-M-027	150, 75, 37.5	100
Protocatechuic acid	99-50-3	Chengdu Chroma-Biotechnology Co., Ltd.	CHB-Y-159	200, 100, 50	200
β-sitostrerol	83-46-5	Chengdu Chroma-Biotechnology Co., Ltd.	CHB-G-056	12, 6, 3	12

* HPLC, MS, and NMR have identified all compounds, and the purity is≥ HPLC 98%.

Western blotting data:

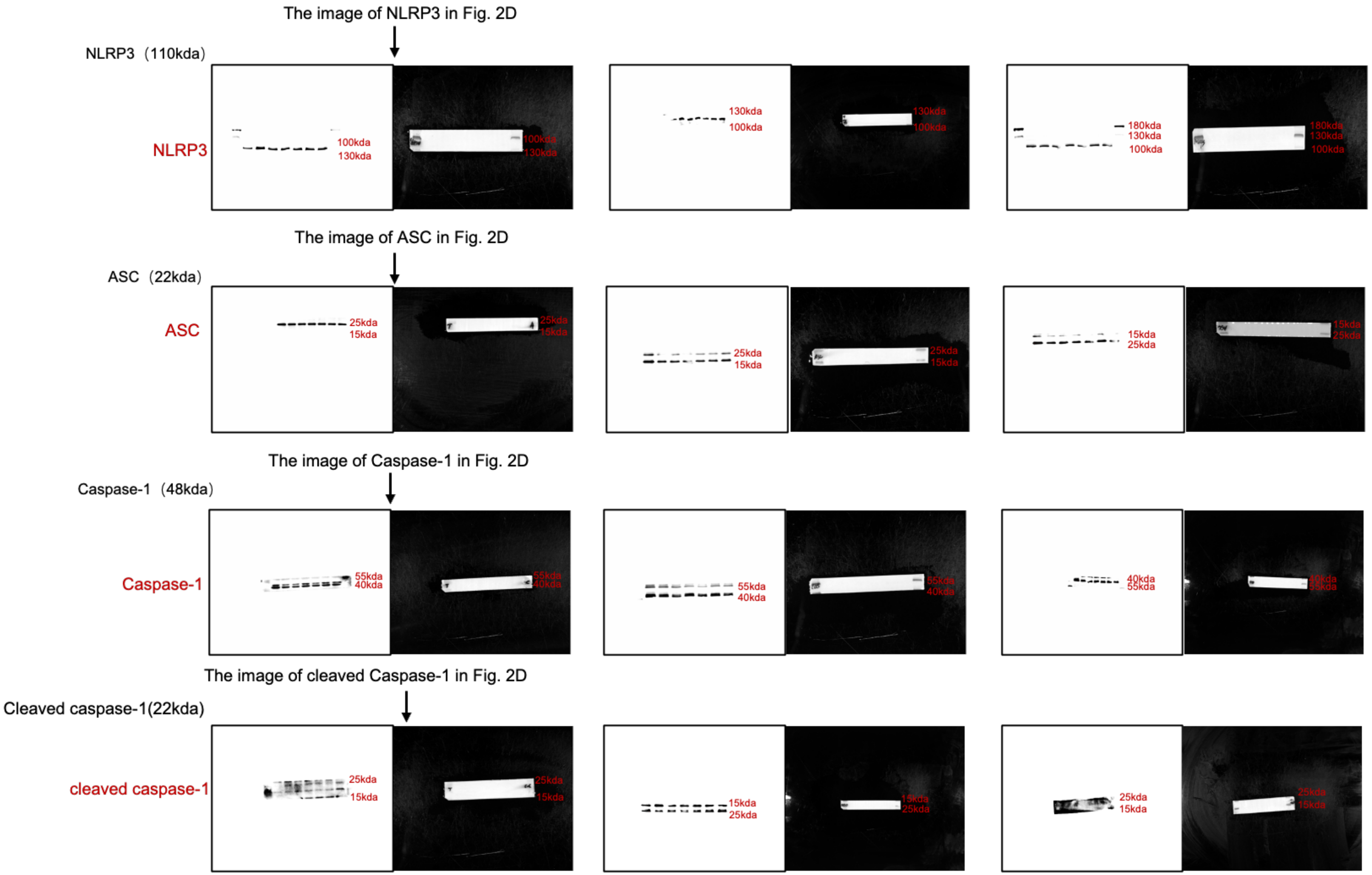

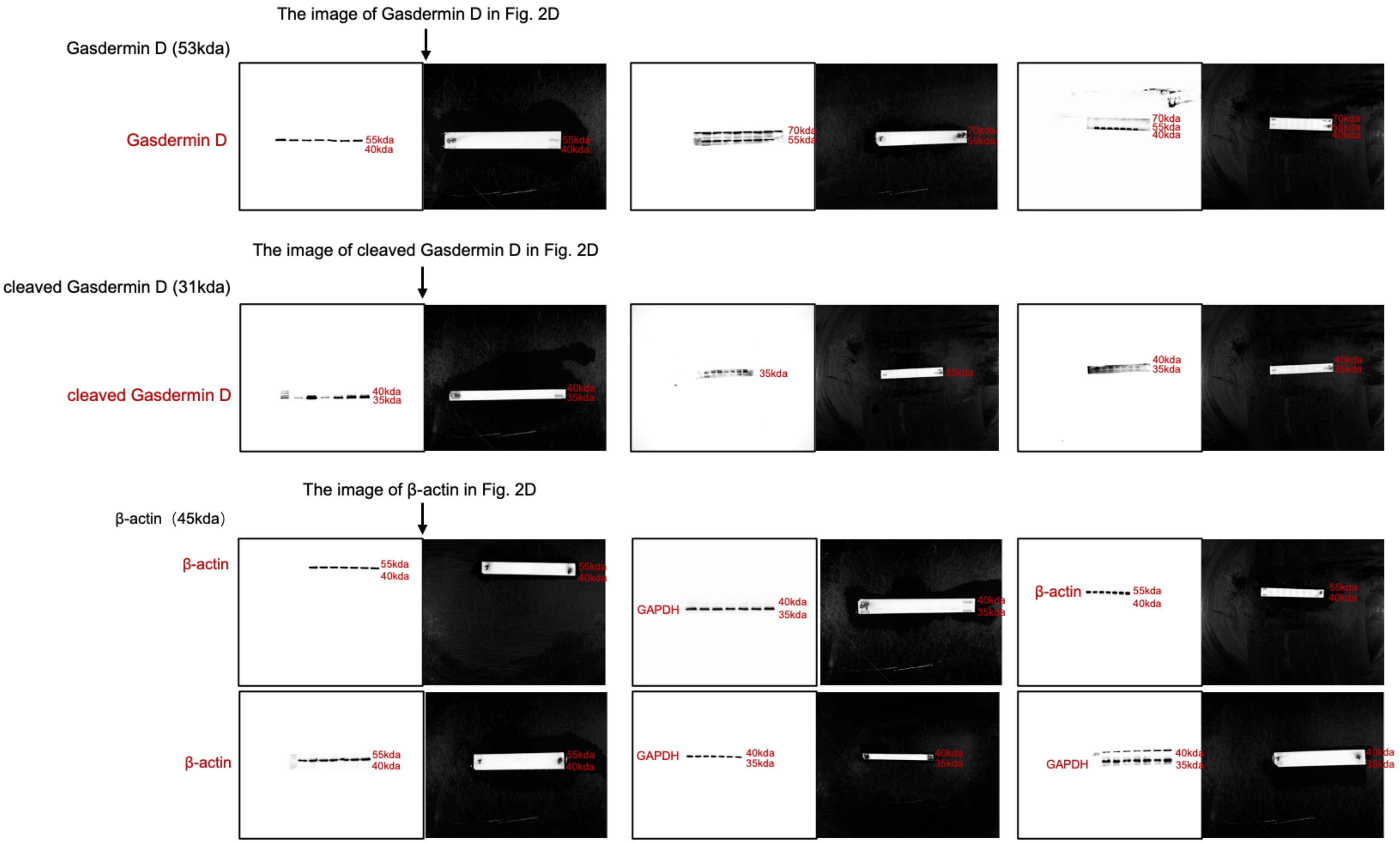
The image of Gasdermin D in Fig. 2D
Gasdermin D (53kda)
Gasdermin D
55kda
40kda
70kda
55kda
70kda
55kda
40kda
The image of cleaved Gasdermin D in Fig. 2D
cleaved Gasdermin D (31kda)
cleaved Gasdermin D
40kda
35kda
35kda
40kda
35kda
The image of β-actin in Fig. 2D
β-actin（45kda）
β-actin
55kda
40kda
GAPDH
40kda
35kda
β-actin
55kda
40kda
β-actin
55kda
40kda
GAPDH
40kda
35kda
GAPDH
40kda
35kda

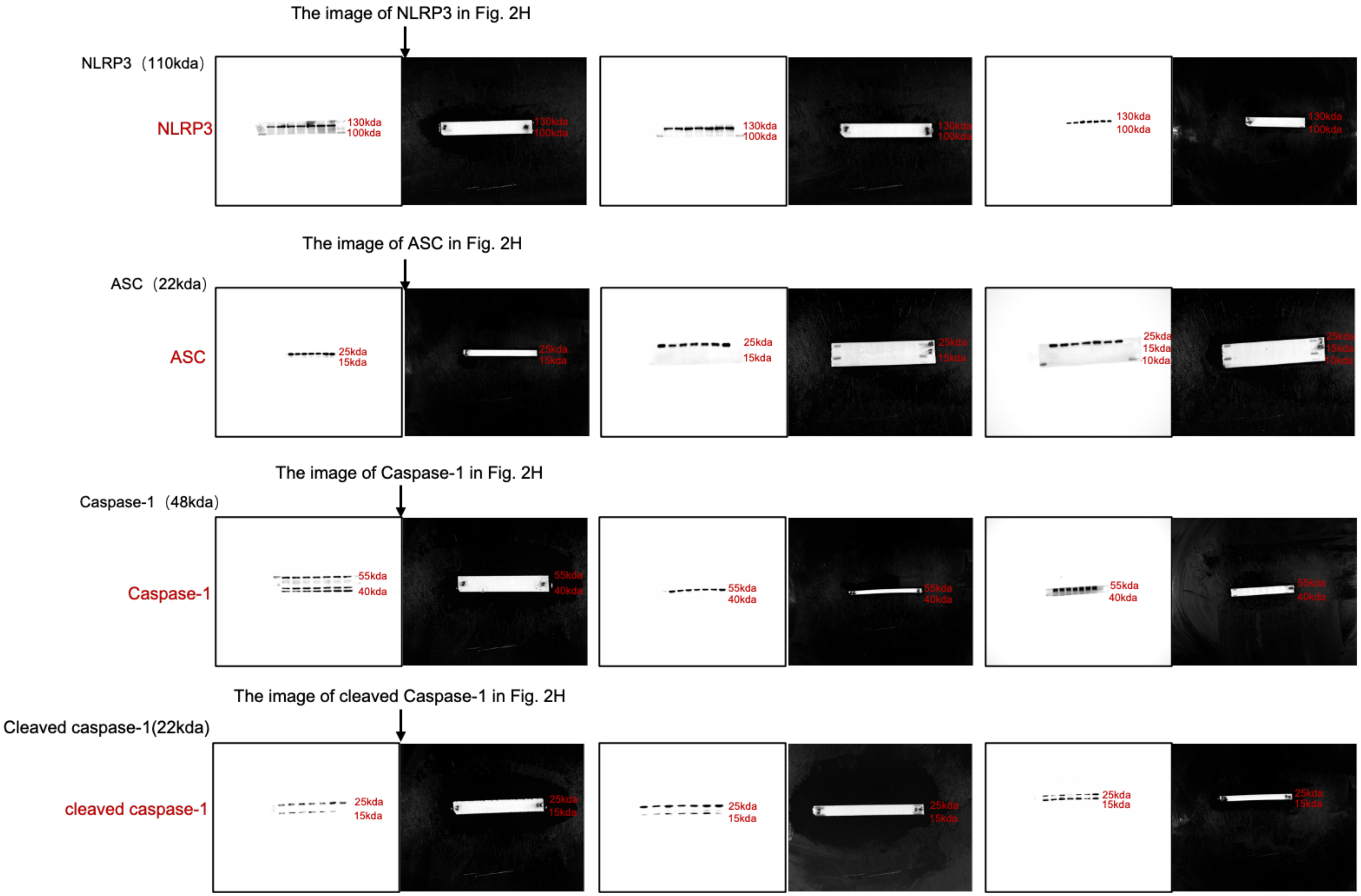
The image of NLRP3 in Fig. 2H
NLRP3（110kda）
NLRP3
130kda
100kda
The image of ASC in Fig. 2H
ASC（22kda）
ASC
25kda
15kda
10kda
The image of Caspase-1 in Fig. 2H
Caspase-1（48kda）
Caspase-1
55kda
40kda
The image of cleaved Caspase-1 in Fig. 2H
Cleaved caspase-1(22kda)
cleaved caspase-1
25kda
15kda

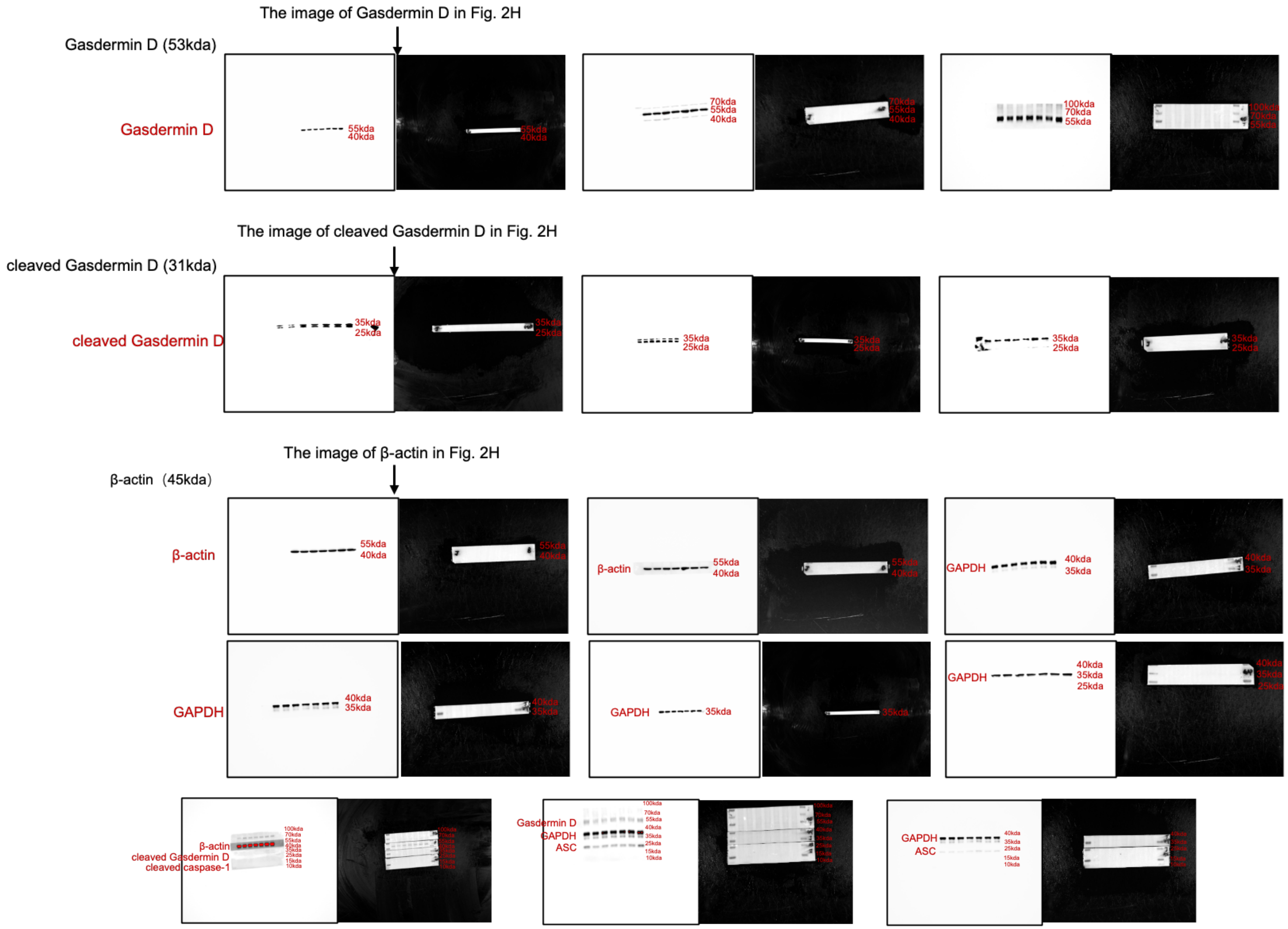
The image of Gasdermin D in Fig. 2H
Gasdermin D (53kda)
Gasdermin D
55kda
40kda
70kda
55kda
40kda
100kda
70kda
55kda
The image of cleaved Gasdermin D in Fig. 2H
cleaved Gasdermin D (31kda)
cleaved Gasdermin D
35kda
25kda
The image of β-actin in Fig. 2H
β-actin（45kda）
β-actin
55kda
40kda
GAPDH
40kda
35kda
25kda
β-actin
cleaved Gasdermin D
cleaved caspase-1
Gasdermin D
GAPDH
ASC

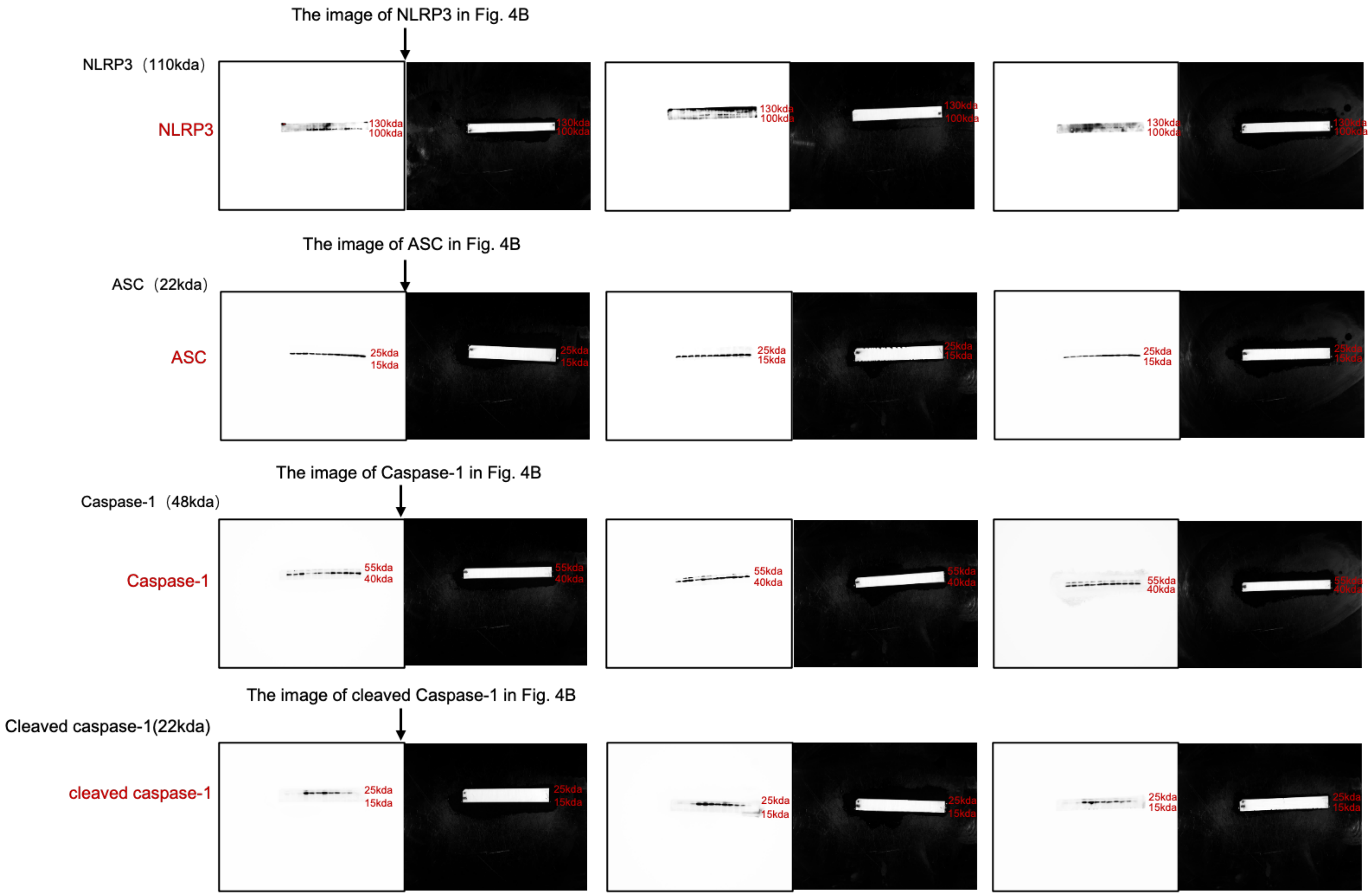

The image of NLRP3 in Fig. 4B
NLRP3（110kda）
NLRP3
130kda
100kda
The image of ASC in Fig. 4B
ASC（22kda）
ASC
25kda
15kda
The image of Caspase-1 in Fig. 4B
Caspase-1（48kda）
Caspase-1
55kda
40kda
The image of cleaved Caspase-1 in Fig. 4B
Cleaved caspase-1(22kda)
cleaved caspase-1
25kda
15kda

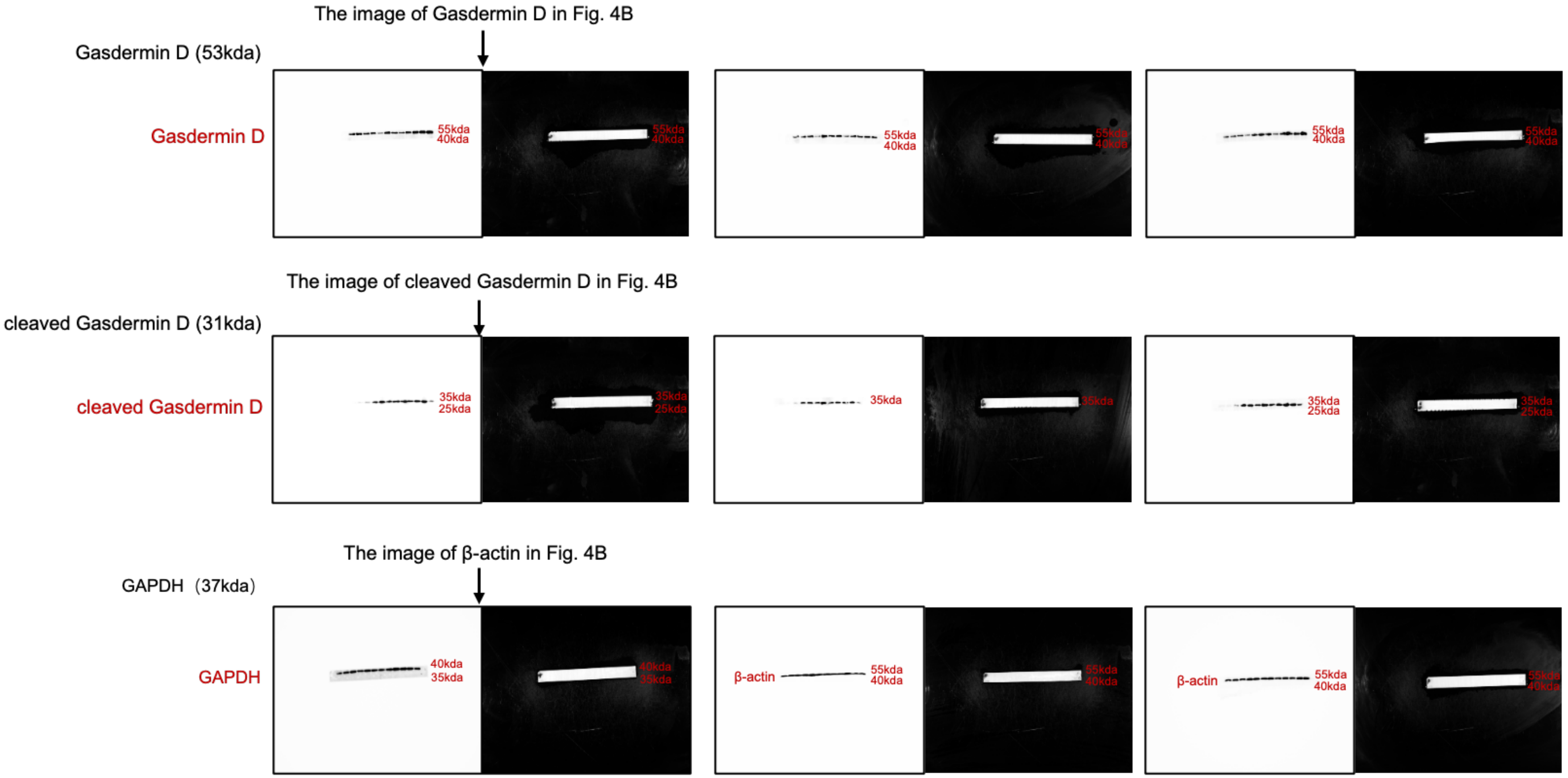

The image of Gasdermin D in Fig. 4B
Gasdermin D (53kda)
Gasdermin D
55kda
40kda
The image of cleaved Gasdermin D in Fig. 4B
cleaved Gasdermin D (31kda)
cleaved Gasdermin D
35kda
25kda
The image of β-actin in Fig. 4B
GAPDH（37kda）
GAPDH
40kda
35kda
β-actin
55kda
40kda

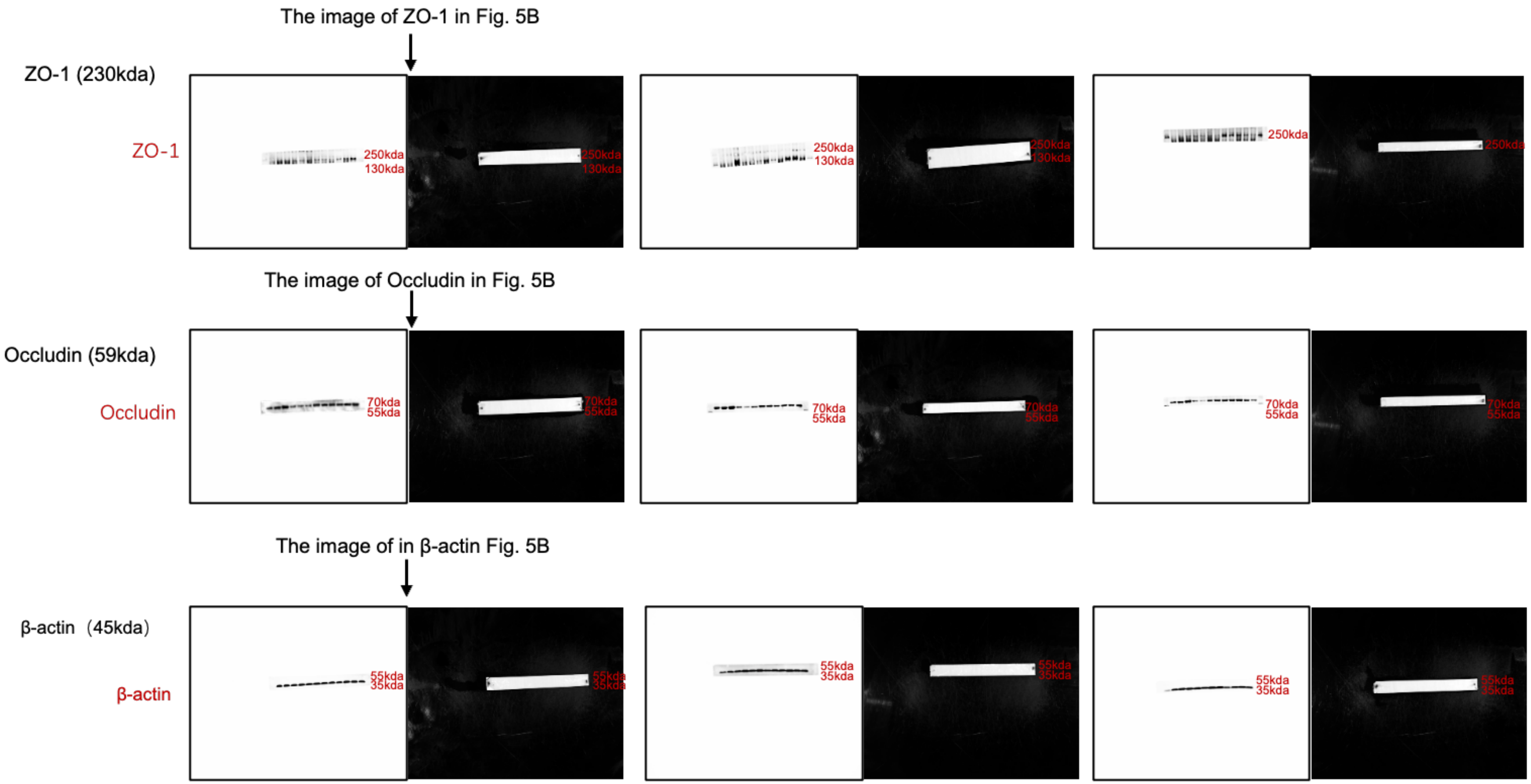
The image of ZO-1 in Fig. 5B
ZO-1 (230kda)
ZO-1
250kda
130kda
The image of Occludin in Fig. 5B
Occludin (59kda)
Occludin
70kda
55kda
The image of in β-actin Fig. 5B
β-actin（45kda）
β-actin
55kda
35kda

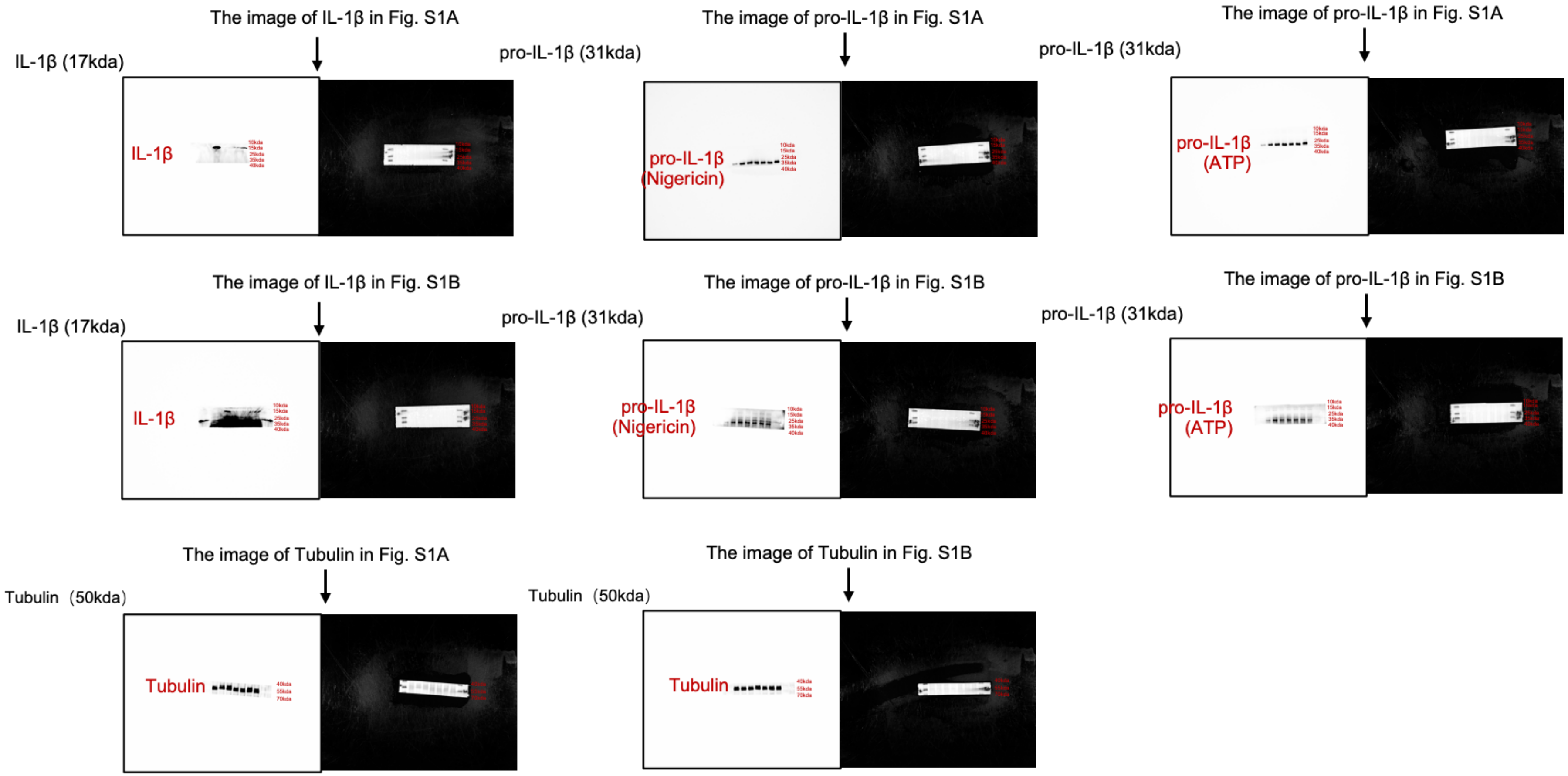

The image of IL-1β in Fig. S1A
IL-1β (17kda)
IL-1β
The image of pro-IL-1β in Fig. S1A
pro-IL-1β (31kda)
pro-IL-1β (Nigericin)
The image of pro-IL-1β in Fig. S1A
pro-IL-1β (31kda)
pro-IL-1β (ATP)
The image of IL-1β in Fig. S1B
IL-1β (17kda)
IL-1β
The image of pro-IL-1β in Fig. S1B
pro-IL-1β (31kda)
pro-IL-1β (Nigericin)
The image of pro-IL-1β in Fig. S1B
pro-IL-1β (31kda)
pro-IL-1β (ATP)
The image of Tubulin in Fig. S1A
Tubulin（50kda）
Tubulin
The image of Tubulin in Fig. S1B
Tubulin（50kda）
Tubulin

The image of IκB in Fig. S2

IκB(39kda)

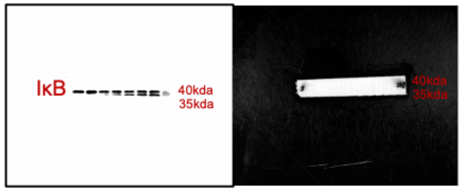

The image of p65 in Fig. S2

p65(65kda)

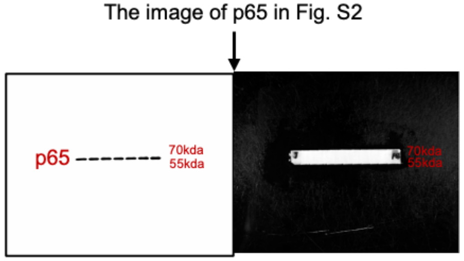

The image of β-actin in Fig. S2

β-actin（45kda）

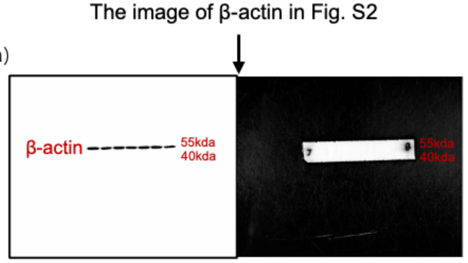

The image of p-IκB in Fig. S2

p-IκB(40kda)

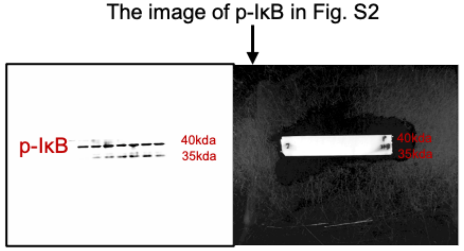

The image of p-p65 in Fig. S2

p-p65(65kda)

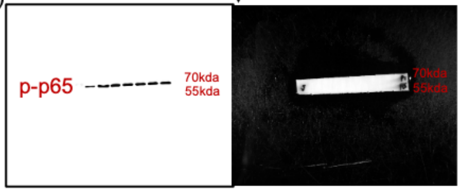

Supplement: Supplementary file 1 [file DataSheet1.pdf]
